# Usefulness of A Portal Vein Stent for Sinistral Portal Hypertension: A Case Report

**DOI:** 10.70352/scrj.cr.25-0206

**Published:** 2025-07-16

**Authors:** Daisuke Takimoto, Jun Ishida, Hirochika Toyama, Yoshihide Nanno, Takuya Mizumoto, Toshihiko Yoshida, Takeshi Urade, Kenji Fukushima, Hidetoshi Gon, Daisuke Tsugawa, Shohei Komatsu, Hiroaki Yanagimoto, Masahiro Kido, Takumi Fukumoto

**Affiliations:** Department of Surgery, Division of Hepato-Biliary-Pancreatic Surgery, Graduate School of Medicine, Kobe University, Kobe, Hyogo, Japan

**Keywords:** pancreatoduodenectomy, portal vein stenosis, splenic vein stenosis, sinistral portal hypertension, portal vein stent

## Abstract

**INTRODUCTION:**

Portal vein (PV) and splenic vein (SV) stenosis are known complications of pancreatoduodenectomy (PD) and often lead to portal hypertension. PV stenosis extending to the SV confluence can result in sinistral portal hypertension (SPH), characterized by gastrointestinal varices and splenomegaly in the presence of normal liver function. There is no standardized treatment strategy for SPH following PD.

**CASE PRESENTATION:**

A 42-year-old female underwent robot-assisted PD for a pancreatic neuroendocrine tumor without immediate PV complications. Postoperatively, the patient experienced fluid retention; however, this did not pose a problem, and no therapeutic intervention was necessary. Two months later, imaging revealed PV stenosis and SV obstruction. Eleven months after surgery, the patient presented with melena, and imaging confirmed the presence of gastroesophageal varices with severe PV stenosis and complete SV obstruction. Endoscopic variceal ligation was performed, and the hemodynamic status of the portal system was assessed using computed tomography during arterial portography (CTAP). CTAP showed communication between the superior mesenteric vein and the SV via the middle colic vein. Therefore, we decided to perform PV stenting. The stent was successfully placed, resulting in a significant improvement in the esophageal varices. The patient was discharged on postoperative day 4, receiving anticoagulant therapy, with no further complications.

**CONCLUSIONS:**

This case demonstrates the efficacy of PV stenting after careful hemodynamic assessment in a patient who developed SPH due to PV stenosis and SV obstruction following PD.

## Abbreviations

CTAP
: computed tomography during arterial portography
IMV
: inferior mesenteric vein
LGV
: left gastric vein
MCV
: middle colic vein
NET
: neuroendocrine tumor
PD
: pancreatoduodenectomy
PV
: portal vein
PVS
: portal vein stenting
SAE
: splenic artery embolization
SPH
: sinistral portal hypertension
SMV
: superior mesenteric vein
SV
: splenic vein
SVS
: splenic vein stenting

## INTRODUCTION

Stenosis of the PV and SV after PD is a widely acknowledged postoperative complication.^1,2)^ This condition can result from postoperative inflammation due to pancreatic leakage, radiotherapy, local cancer recurrence, and concurrent SMV/PV resection, potentially leading to symptoms of portal hypertension.^1,2)^ Recent studies on PVS for PV stenosis have shown a significant reduction in portal hypertension and relief of related symptoms.^3,4)^ However, if the PV stenosis extends to the SV confluence, SPH can occur. SPH is characterized by impaired blood flow from the spleen due to SV obstruction, resulting in gastrointestinal varices and splenomegaly, even in the presence of normal liver function and portal venous flow.^5)^ Treating PV stenosis using SPH is challenging because PVS cannot alleviate SV obstruction.

In the present case, PV stenosis and SV obstruction were caused by fluid retention after robot-assisted PD of an NET. As a result, the patient developed SPH and rupture of the esophageal varices, leading to bleeding. After evaluating the patient's hemodynamic status, PVS was performed, which resulted in a marked improvement in the esophageal varices. Herein, we present this case and discuss the clinical significance of hemodynamic monitoring and treatment using PVS for SPH.

## CASE PRESENTATION

**Fig. 1:**
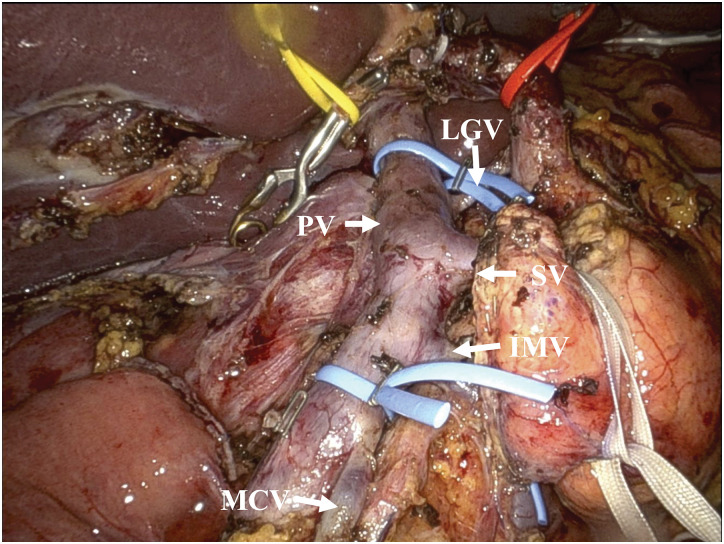
Photograph of the PV immediately after resection. The LGV, MCV, SV, and IMV were preserved. IMV, inferior mesenteric vein; LGV, left gastric vein; MCV, middle colic vein; PV, portal vein; SV, splenic vein

**Fig. 2:**
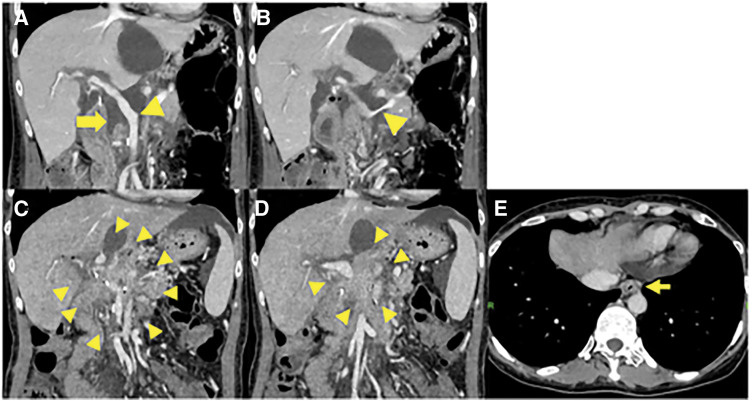
(**A**, **B**) Enhanced CT at 1 week after surgery. Enhanced CT showed fluid accumulation around the PV and SV (arrows). There was mild stenosis but no obvious blood flow obstruction in the PV or SV (arrowhead). (**C–E**) Enhanced CT at 2 months after surgery. (**C**, **D**) Inflammatory changes around the PV and obstruction of the SV were observed (arrowheads). (**E**) No development of esophageal varices (arrow). PV, portal vein; SV, splenic vein

**Fig. 3:**
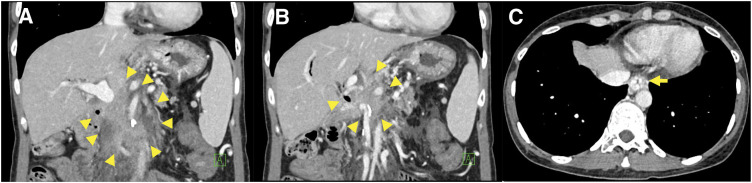
Enhanced CT at the time of hemorrhage. (**A**, **B**) Inflammatory changes were observed around the PV, along with severe stenosis of the PV and obstruction of the SV (arrowheads). (**C**) The development of esophageal varices was noted (arrow). PV, portal vein; SV, splenic vein

**Fig. 4:**
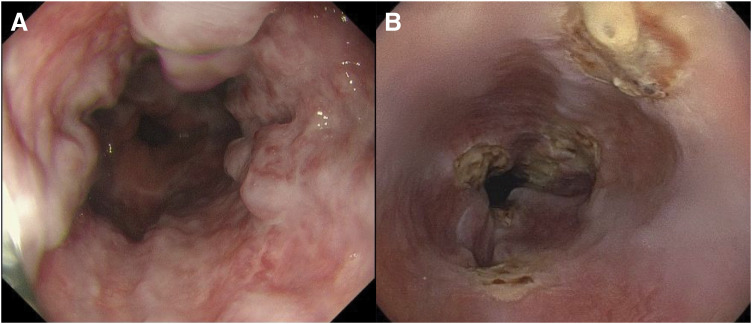
(**A**) Upper gastrointestinal endoscopy at the time of bleeding. Well-developed varices were observed in a circumferential fashion from the incisor 30 cm to the esophagogastric junction. (**B**) Upper gastrointestinal endoscopy 2 days after portal vein stenting. The esophageal varices had nearly disappeared, leaving only scars from endoscopic variceal ligation.

**Fig. 5:**
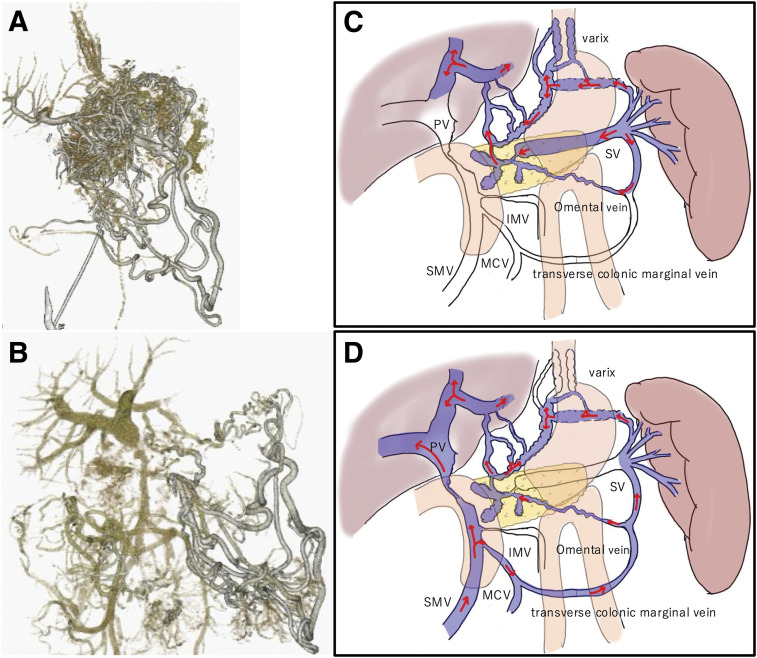
CT during arterial portography. (**A**, **C**) Splenic arteriography revealed obstruction of the SV. Furthermore, the collateral veins around the esophagus and stomach were similarly enhanced, and the left PV was contrasted via the collateral vein around the pancreaticojejunal anastomosis. (**B**, **D**) SMA angiography showed that a portion of the SV, as well as the esophageal and perigastric collateral veins, were contrasted from the MCV via the omental vein and the transverse colonic marginal vein. IMV, inferior mesenteric vein; MCV, middle colic vein; PV, portal vein; SMA, superior mesenteric artery; SMV, superior mesenteric vein; SV, splenic vein

A 42-year-old female with no relevant medical history underwent robot-assisted PD without PV complications for the diagnosis of pancreatic NET. **Figure 1** shows an intraoperative image of the PV immediately after resection. The LGV, MCV, SV, and IMV were preserved (**Fig. 1**). One week after surgery, enhanced CT scans showed fluid accumulation around the PV and SV, with mild stenosis but no obvious blood flow obstruction (**Fig. 2A, 2B**). Additionally, the LGV was obstructed. The patient was diagnosed with postoperative fluid accumulation, which resolved with conservative treatment. She was subsequently discharged from the hospital on postoperative day 17. Two months after surgery, a CT scan showed inflammatory changes around the PV due to PV stenosis and SV obstruction; however, esophageal varices had not developed (**Fig. 2C**–**2E**). As the patient was asymptomatic at that time, observation without intervention was chosen. Eleven months after the surgery, the patient visited our hospital with a chief complaint of melena. A physical examination revealed only conjunctival pallor, and blood tests showed a hemoglobin level of 6.6 g/dL and a hematocrit of 21.4%. CT showed the development of gastroesophageal varices, in addition to complete SV obstruction and severe PV stenosis, similar to the CT findings 2 months postoperatively (**Fig. 3**). Upper gastrointestinal endoscopy revealed esophageal varices. Endoscopic variceal ligation was performed in the erosive and hemorrhagic areas (**Fig. 4A**). Based on these findings, the patient was diagnosed with SPH secondary to PV stenosis and SV obstruction. CTAP was performed to determine the patient’s hemodynamic status. CT imaging after splenic arteriography revealed SV obstruction. Furthermore, the collateral veins around the esophagus and stomach were similarly enhanced, while the left PV was contrasted via the collateral vein around the pancreaticojejunal anastomosis (**Fig. 5A**, **5C**). CT imaging after superior mesenteric arteriography revealed contrast enhancement in a portion of the SV, as well as the esophageal and perigastric collateral veins, originating from the MCV via the omental and transverse colonic marginal veins (**Fig. 5B**, **5D**).

**Fig. 6:**
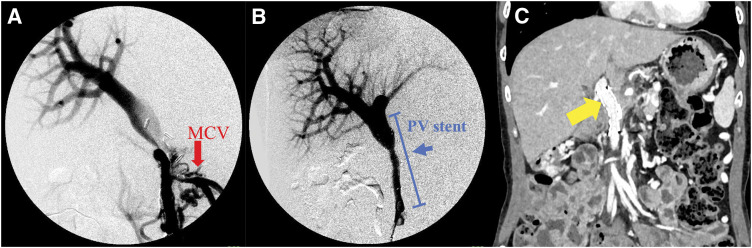
The patient underwent PV stenting via the ileocecal approach. (**A**) Portography before stenting. The left branch of the PV was not contrasted at all, and the MCV was contrasted. (**B**) Portography after stenting. The left branch of the PV was slightly contrasted, and the MCV was no longer contrasted. (**C**) Enhanced CT performed 2 years after PV stenting showed no stent migration or occlusion (arrow) and no recurrence of esophageal varices. MCV, middle colic vein; PV, portal vein

We considered that PV stenting would alleviate PV stenosis and reduce PV pressure, thereby improving SPH, as there is a pathway connecting the SMV and SV via the MCV and colonic marginal vein. The patient underwent PV stenting via an ileocecal approach. A 6-Fr sheath was inserted into the ileocolic vein. Portography revealed PV stenosis and hepatofugal flow toward the MCV due to portal hypertension (**Fig. 6A**). After the stenotic lesions were dilated using balloon catheters, a LUMINEXX stent (1.4–4 cm in diameter; Bard Peripheral Vascular, Tempe, AZ, USA) was implanted. Stent placement resulted in the disappearance of the hepatofugal flow and dilation of the stenosis (**Fig. 6B**). Two days after PV stenting, upper gastrointestinal endoscopy revealed a significant improvement in the esophageal and gastric varices (**Fig. 4B**). Edoxaban was initiated on postoperative day 3 to prevent stent thrombosis, and the patient was discharged on postoperative day 4. She underwent follow-up CT scans for 2 years, which showed no stent migration or occlusion and no recurrence of esophageal varices (**Fig. 6C**).

## DISCUSSION

PV stenosis and the consequent portal hypertension are known complications of PD.^2)^ This condition can result from postoperative inflammation due to pancreatic leakage, radiotherapy, local cancer recurrence, or SMV/PV resection, potentially leading to symptoms of portal hypertension.^1,2)^ The PV stenosis/obstruction rate after PD has been reported to be 19.6%, with a 5-year patency rate of 69.9%. Of these cases, 16.0% and 17.3% were due to benign stenosis and obstruction from thrombotic or postoperative inflammation, respectively, without obstruction from local recurrence. An association between the incidence of benign stenosis after PD and postoperative pancreatic leakage has been reported.^1)^ Despite the absence of a postoperative pancreatic leak, we believe that fluid retention caused the PV stenosis in our case.

In younger patients with low-grade tumors, as in this case, the prognosis tends to be longer, necessitating the consideration of long-term stent safety and patency. Several studies have investigated the long-term patency of PV stents. Stenting for benign PV stenosis, excluding post-liver transplantation and malignant cases, showed a mean patency of 30.1 ± 25.6 months,^3)^ with cumulative patency rates of 79.1% at 6 months and 1 year, and 65.6% at 3 years post-stenting.^6)^ Furthermore, the 5-year PV stent patency rate after liver transplantation is >80%,^7–9)^ and a report on the long-term observations showed that the stent patency rate at 10 years was 75%.^10)^ The long-term patency rate of portal stents for benign PV stenosis appears favorable. Therefore, PV stenting should be considered even in patients expected to have long-term survival, as in this case.

SPH is a rare form of localized portal hypertension resulting from SV thrombosis or obstruction. SPH should be considered in patients with gastrointestinal bleeding, normal liver function test results, and unexplained splenomegaly. When a tumor infiltrates the PV–SMV confluence, the SV is sometimes ligated during PD with vascular resection to achieve a negative margin, resulting in SPH.^11–13)^ Furthermore, postoperative venous collateral flow patterns following SV ligation during PD have been previously studied. Pilgrim et al.^14)^ described 3 cases of PD with SV ligation, confirming the importance of the IMV in decompressing the SV and preventing clinically significant SPH. They also noted that, while maintaining the confluence of the IMV as a decompression pathway from the spleen is important, merely maintaining patency at the SV/IMV junction may not be sufficient to adequately decompress the spleen. Strasberg’s group studied the postoperative hemodynamics in 15 patients who underwent PD with SV ligation and IMV–SV junction resection.^15,16)^ Their findings revealed 2 collateral venous pathways from the spleen: a superior route and an inferior route. The superior route was defined as a pathway originating in the SV, proceeding upward and to the right, and draining into the SMV through the gastric and/or perigastric veins. Conversely, the inferior route was defined as a pathway originating in the SV, proceeding downward to the right, and draining into the SMV through the MCV and/or right colonic marginal vein. In this case, an inferior route was available, but PV stenosis occurred, resulting in variceal rupture. SPH improved with the use of a PV stent to resolve PV stenosis.

SPH is uncommon, with no established consensus on the treatment guidelines. The initial control of variceal bleeding relies on conservative therapies, including balloon tamponade, sclerotherapy, vasoconstriction therapy, and band ligation. When these fail, splenectomy or SAE is typically performed. Splenectomy decompresses the left portal system by interrupting splenic artery flow, whereas SAE offers a less-invasive option for high-risk patients or to reduce intraoperative bleeding.^17–20)^ Recently, intravascular stenting of the SV or PV has emerged as an alternative strategy. Several studies indicate that SVS is associated with lower rebleeding rates than splenectomy or SAE,^21–23)^ and a few case reports have described PVS for SPH.^24)^ To clarify the clinical course, we conducted a review of SPH case reports treated with intravascular stents (**Table 1**).^25–31)^ Including the present report, only 2 cases of PVS for SPH have been reported, with few incorporating a detailed pre-treatment hemodynamic assessment. Our patient had complete SV occlusion with severe stenosis extending to the SV/SMV confluence, rendering SVS unsuitable. Because SV occlusion itself can lead to gastrointestinal bleeding, we performed CTAP to delineate the portal hemodynamics, which demonstrated that PV stenosis was driving the left-sided portal pressure. Guided by these findings, we selected PVS, and post-stenting endoscopy confirmed a marked regression of the varices. This case indicates that when the obstruction involves the SV/SMV confluence, CTAP is valuable for determining whether PVS is the appropriate therapeutic option.

**Table 1:** Summary of case reports of intravascular stenting for SPH

Authors (year)	Age/sex	Etiology	Indication for PV stent	Stent site	Pre-treatment hemodynamic assessment	Symptom resolution
Miao et al. (2025)^25)^	51/M	Autoimmune pancreatitis	GI hemorrhage	SVS	No	Yes
Liang et al. (2024)^26)^	46/M	Chronic pancreatitis	GI hemorrhage	SVS	No	Yes
Yamamoto et al. (2024)^27)^	60/M	Chronic pancreatitis	GI hemorrhage	SVS	No	Yes
Li et al. (2024)^24)^	80/F	Pancreatic tail cancer	GI hemorrhage + ascites	PVS	No	Yes
Füssel et al. (2023)^28)^	58/M	Necrotizing pancreatitis	Colonic hemorrhage	SVS	Yes (splenic arteriography)	Yes
Covello et al. (2021)^29)^	59/M	Necrotizing pancreatitis	Chylous ascites	SVS	No	Yes
El Kininy et al. (2017)^30)^	62/M	Chronic pancreatitis	GI hemorrhage	SVS	No	Yes
Ghelfi et al. (2016)^31)^	68/M	Chronic pancreatitis	GI hemorrhage	SVS	No	Yes
Present case	42/F	Benign stenosis after PD	GI hemorrhage	PVS	Yes (CTAP)	Yes

CTAP, computed tomography arterial portography; F, female; GI, gastrointestinal; M, male; PD, pancreatoduodenectomy; PVS, portal vein stent; SPH, sinistral portal hypertension; SVS, splenic vein stent

## CONCLUSIONS

We encountered a case of SPH caused by benign stenosis of the SV and PV after robot-assisted PD for pancreatic NET. PV stenting was performed, and the esophageal varices improved. Therefore, PV stenting should be considered for SPH with PV stenosis following hemodynamic assessment.

## ACKNOWLEDGMENTS

We thank editage (https://www.editage.jp) for editing a draft of this manuscript.

## DECLARATIONS

### Funding

No grant support or funding from persons or institutions was provided for this case report.

### Authors’ contributions

DTa reported the case and wrote the manuscript.

JI assisted with the composition of the manuscript.

HT supervised this case report.

All authors read and approved the final manuscript.

### Availability of data and materials

The dataset supporting the conclusions of this article is included within the article.

### Ethics approval and consent to participate

This work does not require ethical considerations or approval. Written informed consent was obtained, and the study adhered to ethical standards, respecting patient privacy.

### Consent for publication

Written informed consent for publication of individual data and any accompanying images was obtained from the patient.

### Competing interests

The authors declare no conflicts of interest.
